# The impact of a ketogenic diet on weight loss, metabolism, body composition and quality of life

**DOI:** 10.1016/j.isci.2024.111291

**Published:** 2024-10-30

**Authors:** Simon Hirschberger, David Effinger, Polina Yoncheva, Annika Schmid, Mara-Noel Weis, Lesca-Miriam Holdt, Daniel Teupser, Simone Kreth

**Affiliations:** 1 Walter Brendel Centre of Experimental Medicine, Ludwig-Maximilian-University (LMU) Munich, 81377 Munich, Germany; 2 Department of Anaesthesiology, Research Unit Immune Function and Immune Metabolism, LMU University Hospital, LMU Munich, 81377 Munich, Germany; 3 Institute of Laboratory Medicine, LMU University Hospital, LMU Munich, 81377 Munich, Germany

**Keywords:** Human metabolism, Diet

## Abstract

A ketogenic diet (KD) is increasingly debated as a countermeasure against nutrition-related modern diseases. While being immunologically beneficial, KD is still suspected of having severe metabolic side effects and negatively impacting general well-being, which prevents its widespread clinical use. We conducted a prospective pre-post interventional study investigating the effects of an eucaloric KD on metabolism, weight loss, body composition, diet adherence, and quality of life. The study had two stages: first, feasibility was tested in healthy, normal-weight participants over three weeks. After positive results, the KD period was expanded to three months, enrolling adults with overweight. Significant weight loss was observed in both groups, reducing body fat without affecting muscle or bone mass and without adverse metabolic changes. Quality of life improved, and fatigue symptoms in subjects with overweight decreased. These findings may help to overcome reservations about KD, encouraging its use as a medical tool for treating nutrition-related disorders.

## Highlights

• Weight loss on ketogenic diet is due to visceral fat loss, preserving muscle mass
• Cholesterol levels remain stable during ketogenic diet
• Ketogenic diet enhances general and health-related quality of life
• Fatigue symptoms decrease in overweight individuals on a ketogenic diet

## Graphical abstract

**Figure:**
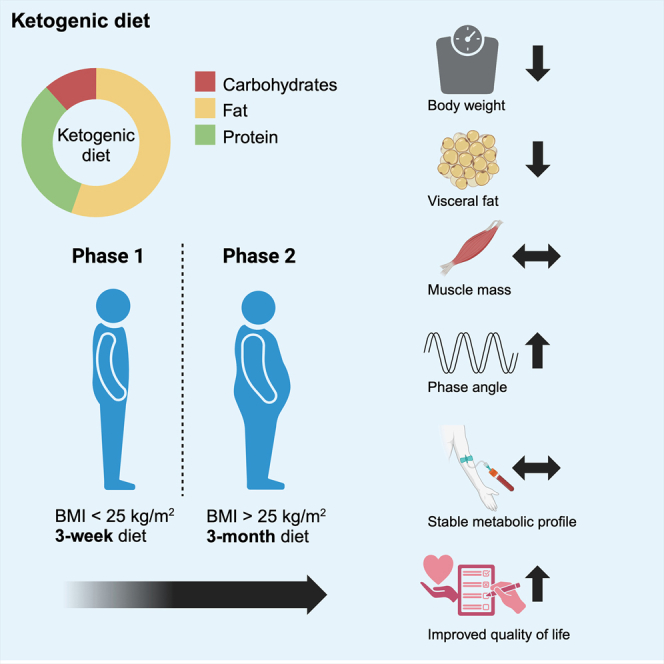

## Introduction

The production and mitochondrial utilization of ketones is an ancient, evolutionarily conserved metabolic strategy that enables humans to survive in times of food scarcity. In a ketogenic metabolic state, the stored body fat is converted hepatically into ketone bodies—mainly β-hydroxybutyrate (BHB)—which efficiently supply the non-hepatic organs with the required energy.^1^^,^^2^ In our modern world, this mechanism has been re-discovered. The reasons for this are of course not a lack of food but rather the opposite: The side effects of the typical carbohydrate-overemphasizing diet of modern societies, namely obesity and the associated systemic low-grade inflammatory state, are taking on pandemic proportions not only driving a plethora of metabolic and cardiovascular diseases but also cancer, neurodegenerative diseases, and depression.^3^^,^^4^ A ketogenic diet (KD), which metabolically mimics the fasting state by drastically reducing carbohydrates in favor of fat, is gaining attention as a potential countermeasure. KD is increasingly discussed as a tool for weight loss and as a potential health promoting intervention.^5^^,^^6^^,^^7^ This idea is based on the fact that KD interrupts the vicious circle of glucose intake and insulin production and enables the “trapped” body fat to be used for ketogenesis. Moreover, positive immunomodulatory properties have recently been found: ketones have been revealed as potent inhibitors of inflammatory processes while adaptive immunity is strengthened.^8^^,^^9^^,^^10^

In view of all these positive aspects, it is surprising that KD is not widely used to treat nutrition-related diseases. Doubts continue to persist, which particularly concern severe metabolic complications of KD, such as hyperlipidemia, unintended loss of fat-free mass, and insufficient dietary adherence due to an impairment of general well-being.^11^ Yet, scientific human studies to substantiate or refute these reservations are still scarce.

To shed new light on this ongoing controversy, we performed a prospective pre-post interventional study, investigating the effects of an eucaloric KD on metabolism, weight loss and body composition, dietary adherence, and quality of life. We monitored dietary success by regular point-of-care quantification of blood BHB. A two-stage study design was used: first, we tested the feasibility of this approach in healthy participants of normal weight over a time period of three weeks. Second, after obtaining positive results, we expanded our approach to a KD period of 3 months enrolling exclusively adults with overweight.

We found positive effects of KD in both intervention groups: significant weight loss was achieved by reducing body fat mass without affecting muscle or bone mass and without adverse metabolic changes. Surprisingly, we also could show that the quality of life of participants on a KD improved. These data may provide important new insights to overcome the reservations about KD and render it suitable as a routine medical tool for treating nutrition-related disorders.

## Results

### Effects of three weeks ketogenic diet

**Figure 1:**
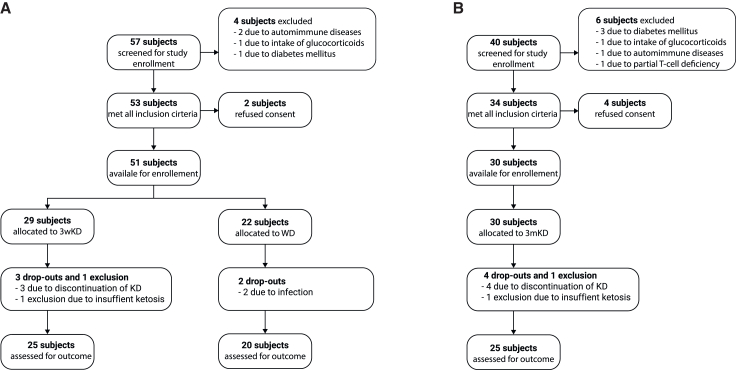
CONSORT recruitment flow diagram (A) CONSORT diagram for dietary study period of three weeks. (B) CONSORT diagram for dietwary study period of three months. 3wKD = three weeks ketogenic diet, 3mKD = three months ketogenic diet, WD = Western diet, KD = ketogenic diet.

First, the effects of three weeks KD (3wKD) were assessed with focus on adherence of participants, impact on metabolism, body composition, and quality of life. 57 volunteers were screened for study eligibility. Of these, 51 were included, with 29 subjects assigned to the 3wKD group and 22 subjects assigned to the Western diet (WD) group. In the 3wKD group, three subjects dropped out during the intervention, and one subject was retrospectively excluded due to insufficient ketogenesis (Figure 1A). No adverse events were reported throughout the study period. The demographic characteristics of participants on a 3wKD as well as mean physical activity level (PAL) and mean caloric intake are depicted in Table 1.

**Table 1:** Baseline characteristics of participants following a three-week KD

	Three-week KD (3wKD)
n	25
Age [years], *mean ±SD*	41.12 ± 13.28
Sex [%], male/female/diverse	25/75/0
BMI, [kg/m2], mean ± SD	22.83 ± 1.92
PAL, *mean ±SD*	1.82 ± 0.12
Calculated caloric intake [kcal/day], *mean ±SD*	2644 ± 271
Calculated carbohydrate intake [g/day], *mean ±SD*	33.06 ± 3.39

**Figure 2:**
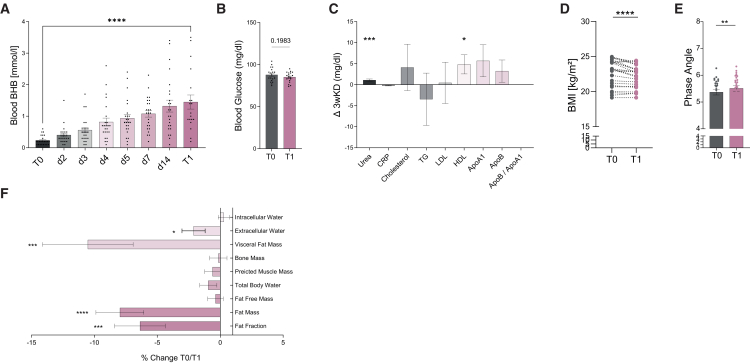
Changes to serum metabolic markers and bioelectrical impedance in response to three-weeks ketogenic nutritional intervention (A and B) (A) Blood ketone body concentration quantified via point-of-care blood ketone strips and (B) fasting blood glucose prior to the start of the diet (T0) and at the end of the diet (T1). (C) Changes to metabolic parameters (T1-T0) as indicated. (D–F) Anthropometric data as indicated, collected via bioelectric impedance analysis prior to the start (T0) and at the end (T1) of three weeks KD. Data depicted as mean ± SEM (A, B, and E), change (mg/dL) T1-T0 (C), % change T1/T0 (F), and symbols and lines (D), with dots indicating individual values. *n* = 25. Paired t test or Wilcoxon matched-pairs signed rank test, as appropriate. Benjamini Hochberg multiple testing correction. ∗*p* < 0.05, ∗∗*p* < 0.01, ∗∗∗*p* < 0.001, ∗∗∗∗*p* < 0.0001, with FDR <10%.

Participants on a KD successfully generated ketone bodies with mean blood BHB levels increasing from 0.24 ± 0.15 mmol/L at the start of the diet (T0) to 1.45 ± 0.95 mmol/L at the end of the diet (T1, *p* < 0.0001). The state of ketosis was achieved within three to five days with mean blood BHB concentrations exceeding 0.5 mmol/L (d3: 0.56 ± 0.40 mmol/L, d4: 0.86 ± 0.61 mmol/L, d5: 0.94 ± 0.59 mmol/L). Ketosis remained stable throughout the whole study period (Figure 2A). Fasting blood glucose remained within a normal range during the evaluation period (T0: 87.8 ± 8.2 mg/dL vs. T1: 85.1 ± 6.0 mg/dL, *p* = 0.1983; Figure 2B).

We next analyzed fasting serum samples of volunteers on a 3wKD (Figure 2C). C-reactive protein (CRP) slightly decreased yet without statistical significance (−0.19 ± 0.45 mg/dL, *p* = 0.0859). We detected a modest elevation of serum urea on a KD (+1.1 ± 1.2 mg/dL, *p* = 0.0006). Cholesterol, low density lipoproteins (LDL)-cholesterol and triglycerides (TG) remained unchanged. Of note, high density lipoproteins (HDL)-cholesterol was significantly increased on a KD (+4.8 ± 9.9 mg/dL, *p* = 0.0471). Cardiovascular risk markers Apo A1, Apo B, and Apo B/Apo A1 ratio did not show alterations. T1 blood BHB concentrations did not correlate to Δ HDL or Δ urea (Table S2).

3wKD moderately reduced body weight and body mass index (BMI, T0: 22.65 ± 1.9 kg/m^2^ vs. T1: 22.18 ± 1.6 kg/m^2^, *p* = 0.0002; Figure 2D) as determined by bioelectric impedance analysis (BIA, Table 2). Weight loss was more pronounced in subjects with higher T0 BMI. The extent of weight loss (Δ BMI) did neither correlate to the extent of ketosis (BHB T1) nor to changes of serum urea, CRP, TG, or HDL (Table S2). Phase angle—serving as an indicator of cellular integrity—increased in response to the diet (Figure 2E). BIA (Figure 2F) revealed that weight loss was mainly attributed to the reduction of fat, as indicated by the decline of fat mass (−7.9% ± 9.5%, *p* < 0.0001) and fat fraction (−6.4% ± 10.1%, *p* = 0.0012). Visceral fat decreased accordingly (−10.8% ± 13.4%, *p* = 0.0005), whereas fat free mass, bone mass, and muscle mass remained unchanged. Calculation of body water distribution showed a significant reduction of extracellular water (T0: 15.21 ± 2.20 kg vs. T1: 14.85 ± 2.00 kg, *p* = 0.0298) with no changes to intracellular water. These data indicate beneficial effects of a 3wKD on the body composition of healthy participants, including the loss of fat mass and the preservation of muscle and bone mass.

**Table 2:** Body composition analysis of participants on a 3wKD

	T0 (mean ± SEM)	T1 (mean ± SEM)	*p* value
n	25	25	–
BMI [kg/m^2^]	22.65 ± 0.38	22.18 ± 0.32	**0.0002**
Fat fraction [%]	23.84 ± 1.17	22.57 ± 1.28	**0.0007**
Fat mass [kg]	15.69 ± 0.99	14.48 ± 0.96	**<0.0001**
Fat free mass [kg]	50.77 ± 1.56	50.53 ± 1.50	0.4945
Phase angle [°]	5.369 ± 0.10	5.514 ± 0.11	**0.0028**
Total body water [kg]	33.74 ± 1.15	33.34 ± 1.04	0.1574
Predicted muscle mass [kg]	48.2 ± 1.48	47.98 ± 1.44	0.4440
Bone mass [kg]	2.65 ± 0.08	2.64 ± 0.08	0.6267
Visceral fat mass [kg]	7.60 ± 0.65	6.78 ± 0.61	**0.0005**
Visceral fat level	4.72 ± 0.65	3.64 ± 0.46	**0.0002**
Extracellular water [kg]	15.21 ± 0.44	14.85 ± 0.40	**0.0298**
Intracellular water [kg]	18.28 ± 0.72	18.31 ± 0.69	0.9285

Short form health survey (SF-36) and World health Organization’s quality of life assessment (WHOQOL-BREF) questionnaires were assessed at baseline and at the end of the nutritional intervention (Table 3). 3wKD significantly improved general health perception in all SF-36 sub-domains. Consistent with these results, physical (+5.66% ± 2.29%, *p* = 0.0194), mental (+8.15% ± 2.09%, *p* = 0.0006), social (+5.57% ± 2.41%, *p* = 0.0229), and environmental health (+4.03% ± 1.90%, *p =* 0.0317) domains of the WHOQOL-BREF likewise enhanced. Evaluation of fatigue symptoms detected no pathological Fatigue Assessment Scale (FAS) scores or detrimental changes to fatigue symptoms upon 3wKD (T0 FAS: 20.91; T1 FAS 18.30; Table 3).

**Table 3:** Assessment of health-related quality of life via WHOQOL-BREF and SF-36 questionnaire in participants on a three-week KD

	Three-week KD	*p* value
FAS (mean ± SEM); *n* = 25		
Fatigue Assessment Scale	−14.26% ± 20.73%	**0.0086**
SF-36 (mean ± SEM); *n* = 25		
Physical functioning	+2.37% ± 1.07%	**0.0469**
Physical role functioning	+9.58% ± 4.04%	**0.0312**
Emotional role functioning	+22.94% ± 8.60%	**0.0312**
Bodily pain	+4.29% ± 1.94%	**0.0469**
Mental health	+8.75% ± 2.54%	**0.0012**
Social role functioning	+6.73% ± 2.04%	**0.0039**
General health perception	+6.40% ± 1.89%	**0.0021**
Vitality	+12.98% ± 4.72%	**0.0134**
WHOQOL-BREF (*mean ±SEM*); *n* = 25		
Physical health	+5.66% ± 2.29%	**0.0194**
Mental health	+8.15% ± 2.09%	**0.0006**
Social relationships	+5.57% ± 2.41%	**0.0229**
Environmental quality	+4.03% ± 1.90%	**0.0317**

### Western diet control

To exclude a possible bias by nutritional counseling, we additionally performed all of these analyses in a cohort of healthy volunteers following an eucaloric WD over a time period of three weeks. No adverse events were reported throughout the study period. Characteristics of participants are depicted in Table S3. As expected, no blood BHB was detectable in this group (Figure S1A). Comprehensive metabolic assessment, bioelectric impedance analyses, and QoL questionnaires revealed no significant changes to any parameter during the course of the WD (Figures S1B and S1C; Tables S4 and S5).

### Effects of three months ketogenic diet

In a second step, we investigated the impact of KD on patients with overweight (BMI >25 kg/m^2^), also with focus on body composition, metabolism, and quality of life. Given the positive results obtained in the feasibility group, the study period was expanded to three months (3mKD), and the applied study protocols were adjusted accordingly. 40 patients were screened for study eligibility, of which 30 were enrolled. Four dropouts occurred due to premature discontinuation of the 3mKD. One subject was excluded due to insufficient BHB levels in consecutive measurements (Figure 1B). No adverse events were reported throughout the study period. Patients’ characteristics are summarized in Table 4.

**Table 4:** Characteristics of overweight patient cohort performing 3mKD

	Three-month KD (3mKD)
N	25
Age [years], *mean ±SD*	50.96 ± 12.60
Sex [%], male/female/diverse	32/68/0
BMI, [kg/m2], *mean ±SD*	30.88 ± 4.12
PAL, *mean ±SD*	1.65 ± 0.07
Calculated caloric intake [kcal/day], *mean ±SD*	2839 ± 527
Calculated carbohydrate intake [g/day], *mean ±SD*	35.49 ± 6.59

**Figure 3:**
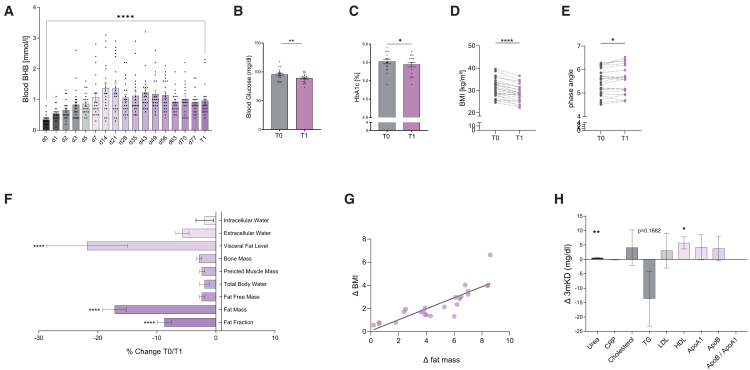
Bioimpedance analysis of patients with overweight following three months KD (A) Blood ketone body concentration quantified via point-of-care blood ketone strips at the time points indicated. (B and C) (B)Fasting blood glucose and (C) HbA1c prior to the start of the diet (T0) and at the end of the three-month KD (T1). (D–F) Anthropometric data as indicated, collected via Bioelectric impedance analysis prior to the start (T0) and at the end (T1) of 3mKD. (G) Correlation analysis of BMI T0-T1 correlated to fat mass T0-T1, with linear regression line displayed. (H) Changes T1-T0 (mg/dL) to the indicated serum metabolic markers in response to nutritional intervention. Data depicted as mean ± SEM (A–C), symbols and lines (D and E), % change T1/T0 (F and H), and change (mg/dL) T1-T0 (H), *n* = 25. Paired t test or Wilcoxon matched-pairs signed rank test, as appropriate. Benjamini Hochberg multiple testing correction. ∗*p* < 0.05, ∗∗*p* < 0.01, ∗∗∗*p* < 0.001, ∗∗∗∗*p* < 0.0001, with FDR <10%.

All patients successfully initiated and maintained ketosis throughout the whole three-month study period (Figure 3A). After the start of the diet (T0: 0.35 ± 0.15 mmol/L), ketosis was detectable within three days (0.83 ± 0.53 mmol/L), reached a maximum level after two weeks (1.37 ± 0.82 mmol/L), and was persistently detectable until the end of the study (T1: 0.96 ± 0.52 mmol/L, T1 vs. T0 *p* < 0.0001). Fasting blood glucose remained within standard range yet decreased at T1 (T0: 95.3 ± 10.1 mg/dL vs. T1: 89.2 ± 7.85 mg/dL, *p* = 0.0048; Figure 3B). HbA1c was also slightly reduced (T0: 5.524% ± 0.256% vs. T1: 5.441 ± 0.269%, *p* = 0.0256; Figure 3C). After three months of KD, significant weight loss occurred (T0: 92.12 ± 3.28 kg vs. T1: 86.28 ± 2.98 kg, *p* < 0.0001), while phase angle increased (T0: 5.453 ± 0.5563 vs. T1: 5.527 ± 0.6037, *p* = 0.0369; Figure 3E). The reduction of BMI did not correlate to the extent of ketosis (r = 0.035, *p* = 0.86) but was strongly correlated to the loss of fat (r = 0.8541, *p* < 0.0001; Figure 3G). Consequently, patients’ body composition fundamentally changed after three months of KD (Table 5; Figure 3F). Fat fraction (−7.6% ± 4.4%), fat mass (−14.3% ± 7.8%), and visceral fat mass (−14.7% ± 12.1%) were markedly reduced, whereas both muscle and bone mass remained unchanged. We detected a decreased fraction of extracellular water (T0: 18.96 ± 2.76 kg vs. T1: 18.00 ± 2.85 kg, *p* < 0.0001), whereas intracellular water remained unaltered (T0: 21.60 ± 1.191 kg vs. T1: 21.29 ± 1.191 kg, *p* = 0.4222).

**Table 5:** Body composition of patients with overweight on a three-month KD

	T0	T1	*p* value
N	25	25	–
Fat fraction [%]	34.76 ± 1.156	32.12 ± 1.174	**<0.0001**
Fat mass [kg]	32.50 ± 1.569	27.85 ± 1.367	**<0.0001**
Fat free mass [kg]	59.46 ± 2.151	58.12 ± 2.163	**<0.0001**
Phase angle [°]	5.453 ± 0.1214	5.527 ± 0.1317	**0.0369**
Total body water [kg]	39.76 ± 1.630	39.09 ± 1.664	0.0873
Predicted muscle mass [kg]	56.48 ± 2.051	55.22 ± 2.062	**<0.0001**
Bone mass [kg]	3.042 ± 0.1060	2.983 ± 0.1063	**<0.0001**
Visceral fat mass [kg]	13.93 ± 1.298	11.89 ± 1.061	**<0.0001**
Visceral fat level	10.13 ± 0.8771	8.625 ± 0.7844	**<0.0001**
Extracellular water [kg]	18.96 ± 0.5518	18.00 ± 0.5691	**<0.0001**
Intracellular water [kg]	21.60 ± 1.191	21.29 ± 1.191	0.4222

Clinically relevant metabolic side effects after 3mKD were not seen (Figure 3H): CRP showed a small reduction yet failed to reach statistical significance (−0.11 ± 0.51 mg/dL, *p* = 0.3185). Blood concentrations of cholesterol, LDL-cholesterol, and triglycerides remained unaltered. Of note, HDL-cholesterol was significantly increased on a 3mKD (+5.7 ± 10 mg/dL, *p* = 0.0123). Cardiovascular risk markers Apo A1, Apo B, and Apo B/Apo A1 ratio depicted no changes on the diet. A slight elevation of serum urea was observed (+0.52 ± 0.88 mg/dL; *p* = 0.0064). Serum metabolic changes did not correlate to the reduction of BMI or to the level of blood ketone bodies (Table S6). These data corroborate the metabolic safety of 3mKD in patients with overweight.

The impact of 3mKD on QoL in patients with overweight was assessed using patient-reported SF-36 and WHOQOL-BREF questionnaires (Table 6). 3mKD markedly improved physical functioning (+8.03% ± 2.04%, *p* < 0.0002), physical role functioning (+14.81% ± 6.91%, *p* = 0.0312), bodily pain (+114.73% ± 4.77%, *p* = 0.0078), mental health (+21.30% ± 5.59%, *p* < 0.0002), social role functioning (+15.33% ± 7.28%, *p* = 0.0371), general health perception (+11.11% ± 4.17%, *p* = 0.0133), and vitality (+35.63% ± 8.66%, *p* < 0.0002). WHOQOL-BREF also revealed improved perception of physical (+13.91% ± 4.11%, *p* = 0.0026) and mental health (+13.57% ± 3.95%, *p* = 0.0023).

**Table 6:** Assessment of health-related quality of life via WHOQOL-BREF and SF-36 questionnaire in participants of three-month KD

	Three-month KD	*p* value
SF-36 (mean ± SEM), *n* = 25		
Physical functioning	+8.03% ± 2.04%	**0.0002**
Physical role functioning	+14.81% ± 6.91%	**0.0312**
Emotional role functioning	+20.00% ± 9.02%	0.0625
Bodily pain	+14.73% ± 4.77%	**0.0078**
Mental health	+21.30% ± 5.59%	**0.0002**
Social role functioning	+15.33% ± 7.28%	**0.0371**
General health perception	+11.11% ± 4.17%	**0.0133**
Vitality	+35.64% ± 8.66%	**0.0002**
WHOQOL-BREF, (mean ± SEM), *n* =25		
Physical health	+13.91% ± 4.11%	**0.0026**
Mental health	+13.57% ± 3.95%	**0.0023**
Social relationships	+7.07% ± 2.95%	**0.0262**
Environmental quality	+4.19% ± 1.64%	**0.0180**

**Figure 4:**
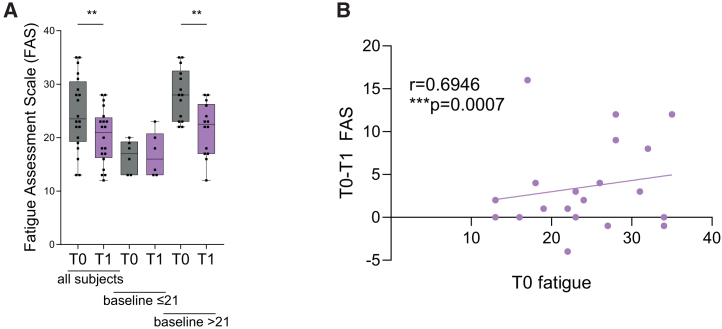
Evaluation of fatigue symptoms of patients with overweight on a KD (A) Fatigue Assessment Scale (FAS), depicted for patients with overweight conducting 3mKD, T0 = at the start of the diet, T1 = at the end of the diet. Data depicted as boxplot with median, 25th and 75th percentiles with dots representing individual values, *n* = 20. Paired t test or Wilcoxon matched-pairs signed rank test, as appropriate. Benjamini Hochberg multiple testing correction. ∗*p* < 0.05, ∗∗*p* < 0.01, with FDR <10%. (B) Correlation analysis of FAS T0-T1 correlated to FAS T0, with linear regression line displayed.

To evaluate overweight-associated fatigue symptoms in this cohort of patients, FAS scores were collected. Three months of KD significantly reduced FAS scores in patients with overweight (−17.89% ± 5.58%, *p* = 0.0046; Figure 4A). This effect was mainly driven by patients exhibiting elevated FAS T0 baseline scores (−22.91% ± 5.92%, *p* = 0.0020). Improvement of FAS was positively correlated to the T0 fatigue level (r = 0.6946, *p* = 0.0007; Figure 4B).

## Discussion

KD is increasingly being considered as a metabolic countermeasure against the nutrition-related diseases of modern societies. While positive effects of KD on the human immune system have recently been proven, the “metabolic landscape” of this diet in the human organism and its influence on general well-being and performance are still debated.^9^^,^^12^^,^^13^ We here show that eucaloric KD is a feasible and effective means of losing weight by reducing fat mass, without reduction of muscle or bone mass, and without harmful metabolic changes, while actually improving quality of life during the period of dietary intervention.

Due to fundamental changes in eating habits, the implementation of KD is considered highly demanding, and the validity of study results could be impaired by a lack of compliance of the participants.^14^ Previous studies simply relied on phone calls or questionnaires to assess adherence to the diet,^15^^,^^16^^,^^17^ and, if at all, only used semi-quantitative urine analyses measuring acetoacetate instead of BHB to detect ketosis.^18^

To overcome these fundamental issues, we have embarked on a comprehensive approach here. All our study participants received individual and intensive nutritional counseling, dietary adherence was closely and objectively monitored by point-of-care blood ketone level quantification, and the dietary impact on metabolism, weight loss, body composition, and quality of life was investigated. In our study cohorts, implementation of KD resulted in a stable generation of sufficient BHB levels, which enabled a scientific investigation of a truly sustained KD over a period of up to three months. A counseling bias could be ruled out by independent analysis of a control group that followed a standard Western diet for three weeks instead of KD but otherwise was treated equally (i.e., counseling, blood sampling, and body analysis).

First, we recruited healthy volunteers of normal weight that were advised to follow an eucaloric KD for three weeks (3wKD). This approach proved feasible and had positive effects on metabolism, body composition, and quality of life. Encouraged by these results, we extended our approach to a second cohort, consisting of patients suffering from overweight that conducted a three-month KD (3mKD) aiming at weight loss.

In both cohorts, serum cholesterol, LDL, and markers of cardiovascular risk—Apo A1 and Apo B—remained stable, whereas beneficial HDL-cholesterol even increased. This may at least partially be mediated by low serum insulin during the diet, as insulin is a central regulator of lipid metabolism, inhibiting β-oxidation of lipids and stimulating cholesterol biosynthesis.^19^^,^^20^ The slight increase in serum urea levels when following a ketogenic diet was probably due to increased protein metabolism, as protein intake is slightly elevated on a KD compared to a conventional diet.^21^

The ability of short-term KD to induce weight loss in patients with overweight has recently been reported;^17^^,^^22^ however, long-term data are still lacking. In addition, it is often assumed that dietary success comes at the cost of a detrimental loss of muscle and bone mass.^12^^,^^23^ In our study, we were able to detect a slight weight loss also in the participants of normal weight of the 3wKD group. Considerable weight loss was observed in all patients with overweight of the 3mKD group. This loss was due to a reduction of fat mass and—especially—visceral fat, while both muscle and bone mass remained stable. Notably, weight and fat loss were positively correlated with the BMI at T0. This fits very well with the theory that on a KD body or dietary fat is converted into BHB and thus ATP, while muscle protein is spared as long as the protein intake is sufficiently high. The reduction in extracellular water, while intracellular water levels remain stable, may indicate an increase in ketone-associated diuresis, but could also reflect reduced inflammation-related water retention.^24^ The mechanistic effect of KD-associated weight loss, however, is still under debate. A reduction in appetite due to the higher satiety effect of proteins and the suppression of appetite-stimulating hormones like ghrelin has been suggested.^25^^,^^26^ However, in our study, the sole change of macronutrient composition without caloric restrictions resulted in weight loss and reduction of body fat. Apparently, being overweight is determined by additional factors beyond energy intake and calorie consumption, indicating that additional explanations for the underlying mechanisms should be considered. The ingestion of fast-acting carbohydrates and the subsequently generated insulin peaks are assumed as one of the further key factors driving weight gain via metabolic shifting toward energy storage.^27^ The reduced glycemic load on a KD may re-program these detrimental metabolic responses. Accordingly, we found significantly reduced HbA1c on a long-term KD. As a third possible explanation, improved food quality during KD may also play a role. Most convenience foods are high in carbohydrates and thus incompatible with KD. Study participants are therefore required to use mainly unprocessed products to prepare their meals, which is generally viewed as a high-quality diet enabling weight loss.^28^^,^^29^^,^^30^

The overall positive effects of KD on body composition were further supported by a significantly higher phase angle (PhA). Initially seen as a marker of cell membrane integrity and body cell mass, PhA has evolved as an objective parameter to assess health and nutritional status. PhA reductions occur in response to diseases, inflammatory processes, and malnutrition, while an increase of PhA indicates health improvements.^31^^,^^32^

To this day, prejudices persist that KD has a negative impact on quality of life and performance. A recently published meta-analysis was not able to get rid of this negative connotation, as the included studies were highly heterogeneous regarding caloric intake, the applied test procedures, etc., and—most importantly—did not stringently control for nutritional ketosis.^13^ We, here, used validated QoL questionnaires (SF-36 and WHOQOL-BREF and FAS) under the condition of controlled eucaloric nutrition with stable ketosis objectively assessed by point-of-care measurements. We could show that participants’ general- and health-related quality of life significantly improved after three weeks of KD. No evidence for a deterioration of social relationships or environmental quality was reported. In the independent cohort following KD for three months, these effects were even more pronounced. Whether these positive effects are directly attributed to the nutritional intervention itself or rather represent an indirect effect of weight loss and fat loss cannot be conclusively clarified here. In addition, we found a considerable reduction of fatigue symptoms among patients of the 3mKD group, which was particularly pronounced among patients initially presenting with higher FAS scores. Fatigue is known to be closely related to obesity^33^^,^^34^ and has also been associated with systemic low-grade inflammatory processes, elevated proinflammatory cytokines, and metabolic dysregulations.^35^^,^^36^^,^^37^ KD may counteract these detrimental effects via direct inhibition of pro-inflammatory pathways and beneficial metabolic reprogramming of immune cells.^8^^,^^9^ Thus, both reduction of overweight and immunological improvements may result in the observed attenuation of fatigue.

Overall, we here show that a ketogenic diet is a feasible and safe nutritional intervention within a three-month time frame to achieve weight loss, improve quality of life, and mitigate overweight-related fatigue. In addition, weight loss even in healthy participants with normal weight may suggest a potential preventive use of KD. The effects of KD on body weight and metabolism beyond the study period of three months remain unclear. In addition, effective strategies for the long-term maintenance of KD-induced weight loss need to be identified. In this context, it is conceivable that intermittent KD phases, alternated with times of less stringent carbohydrate reduction, could be useful. However, further studies are required to clarify these questions.

### Limitations of the study

Our study design has some limitations. As participants interested in the KD were mostly not willing to be assigned to the WD control group, randomization was not feasible. The study was not designed to directly compare WD and KD, but to assess the changes upon dietary intervention on each patient’s individual level. The control group was established to rule out a possible bias caused by the close dietary counseling and monitoring process. Blinding of participants or patients could not be performed due to the nature of the intervention. The high T0 BMI of patients with overweight may create a strong intrinsic motivation to achieve weight loss during the study period. However, the dietary performance (T1 BHB level) did not correlate to the extent of BMI reduction. The enrollment of more female volunteers in phase one may be due to women’s greater interest in nutrition and dietary approaches.^38^ In phase 2, the gender distribution was more balanced, most likely due to an equal motivation of male and female patients with overweight to reduce weight.

## Resource availability

### Lead contact

Further information and requests for resources and reagents should be directed to and will be fulfilled by the lead contact, Prof. Simone Kreth (simone.kreth@med.uni-muenchen.de).

### Materials availability

This study did not generate new unique reagents.

### Data and code availability

• All data reported in this paper will be shared by the lead contact upon request
• This paper does not report original code.
• Any additional information required to reanalyze the data reported in this paper is available from the lead contact upon request.

## Acknowledgments

This work has been supported by institutional grants of the 10.13039/501100005722LMU Munich. S.H. has been supported by the Munich Clinician Scientist Program (MCSP, “FöFoLe+”, #024, LMU Munich). We thank Katja Gieseke, Florian Gosselin, and Bärbel Reincke for their excellent technical assistance. We thank Kristian Unger for his valuable bioinformatic support. The graphical abstract has been created in BioRender (BioRender.com/v51m707).

## Author contributions

Conceptualization, S.H., D.E., and S.K.;methodology, S.H., D.E., and L.-M.H.;validation, S.H., D.E., and L.-M.H.;formal analysis, S.H. and D.E.;investigation, P.Y., A.S., S.H., D.E., M.-N.W., L.-M.H., and D.T.;resources, L.-M.H., D.T., and S.K.;data curation, S.H., D.E., and S.K.;writing original draft, S.H., D.E., and S.K.;visualization, S.H. and D.E.;supervision, S.H., D.E., and S.K.;project administration, S.H., D.E., and S.K.

## Declaration of interests

The authors declare no competing interests.

## STAR★Methods

### Key resources table

**Table:** 

REAGENT or RESOURCE	SOURCE	IDENTIFIER
**Critical commercial assays**		

Roche UREAL kit	Roche Diagnostics, Penzberg, Germany	# 05171873 190
Roche GLUC3 kit	Roche Diagnostics, Penzberg, Germany	# 05168791 190
Roche CHOL2 kit	Roche Diagnostics, Penzberg, Germany	# 05168538 190
Roche TRIGL kit	Roche Diagnostics, Penzberg, Germany	# 05171407 190
Roche HDLC4 kit	Roche Diagnostics, Penzberg, Germany	# 07528582 190
Roche LDLC3 kit	Roche Diagnostics, Penzberg, Germany	# 07005768 190
Roche CRP4 kit	Roche Diagnostics, Penzberg, Germany	# 07876424 190
Roche APOAT kit	Roche Diagnostics, Penzberg, Germany	# 03032566 122
Roche APOBT kit	Roche Diagnostics, Penzberg, Germany	# 03032574 122
Variant II Turbo HvA1c kit-2.0	Bio Rad, Hercules, CA, USA	# 270-2455EX

**Software and algorithms**		

GraphPad Prism 10	GraphPad Software, Boston, MA, USA	https://www.graphpad.com/

**Other**		

Glucomen Aero 2K	Berlin Chemie AG, Berlin, Germany	https://www.glucomenareo.de/
Tanita MC-780	Tanita Europe, Stuttgart, Germany	https://tanita.de/mc-780ma-p
Soehnle Professional Length Measuring Rod 5003	Soehnle GmbH, Backnang, Germany	# 5003.01.001
Variant II Turbo hemoglobin test system	Bio Rad, Hercules, CA, USA	# 270-2601
Roche Cobas 8000/c702 system	Roche Diagnostics, Penzberg, Germany	https://diagnostics.roche.com/us/en/products/instruments/cobas-c-702-ins-2177.html
Cobas 8000/c502 system	Roche Diagnostics, Penzberg, Germany	https://diagnostics.roche.com/us/en/products/instruments/cobas-c-502-ins-2113.html

### Experimental model and study participant details

We investigated the effects of KD on weight loss and body composition, dietary adherence, metabolism, quality of life and fatigue in a prospective pre-post interventional study. The data presented here is part of an experimental nutritional intervention study, for which changes in mRNA expression of IFNγ in T cells was set as the primary endpoint. Secondary endpoints included changes in serum metabolites, body composition, and quality of life. Beyond that, analyzes not prespecified were considered exploratory. All data were collected at our laboratory facilities at the LMU University Hospital, Munich between April 2022 and April 2024. A two-stage study design was used:

First, we tested the feasibility of KD in healthy volunteers of normal weight over a time period of three weeks. Additionally, to control for a possible bias caused by the dietary counseling and monitoring process, we enrolled an additional independent cohort of healthy volunteers that were advised to follow an eucaloric Western Diet (WD) for three weeks. Both groups were comparable with respect to sample size, age and body mass index (BMI), and underwent the same monitoring process.

Second, in case the applied KD protocol proved feasible, and a counseling bias could not be detected, we planned to expand our approach to a KD period of 3 months enrolling exclusively adults with overweight.

Written informed consent was obtained from all volunteers enrolled in the respective study. Research was performed according to the Declaration of Helsinki (ethical principles for medical research involving human subjects). The study designs and the study protocols were approved by the Institutional Ethics Committee of the Ludwig-Maximilian-University Munich, Germany (No. 19–523). Both studies are part of a comprehensive experimental and translational study plan that was registered at the DKRS (German Clinical Trials Register; DRKS-ID: DRKS00027992).

#### Study protocols

##### Dietary period of three weeks

Individuals >18 years were eligible for the study. Exclusion criteria comprised BMI <25 kg/m^2^, women during pregnancy and lactation, and volunteers with current intake of glucocorticoids, severe metabolic disorders (e.g., diabetes mellitus), autoimmune, hematological, or immunological diseases. These criteria were verified by comprehensive and structured medical history interviews and physical examinations.

All study participants received nutritional counseling by board-certified nutritional physicians and nutritionists. Assignment to the KD or WD group was based on the subjects' decision without randomization. The recommended daily caloric intake was based on the individual basal metabolic rate, calculated by the Harris-Benedict-Equation, adjusted to the respective physical activity level of each participant, which was defined through a comprehensive nutritional-medical anamnesis (Table S1).

Participants following a KD were advised to restrict the intake of carbohydrates to less than 10% and to increase fat ingestion to approximately 60% of the daily dietary energy. No further limitations to the individual diet were applied. To evaluate dietary adherence, blood ketone body concentrations were closely monitored using a point of care test device (Glucomen *Aero* 2K; Berlin Chemie AG, Berlin, Germany). For the first week, blood BHB concentrations were measured daily. During the last two weeks of the study period, monitoring was performed twice a week and on the last day of the diet. A ketogenic state (BHB >0.5 mmol/L) was expected to be achieved latest on the beginning of week 2 (day 8). Macronutrient and caloric intake were monitored using food diaries over several days. In cases of insufficient increase in BHB levels the diet was adjusted accordingly. If adequate blood ketone levels could not be obtained, the respective participant was excluded from the study. Baseline characteristics are depicted in Table 1.

Study participants following the WD also received detailed nutritional counseling from a board-certified nutritionist before the start of the study and were closely monitored throughout the entire study period. Participants were counseled according to the “recommendations for a wholesome diet” of the German Council for Nutritional Medicine (DGE). Further details of the counseling (“ten rules for a wholesome diet” of the DGE) are specified in the Supplemental Methods. The macronutrient composition of the WD control group was adhered to the guidelines of the DGE, with caloric intake derived from 50 to 60% carbohydrates, 30% fat and 10–20% protein. To assess the true nutrient uptake and hence caloric and macronutrient uptake of each individual on the WD, 24-h diet recalls by board-certified nutritionists were performed. Baseline characteristics of the participants following a WD are shown in Table S3.

##### Dietary period of three months

In this cohort, individuals >18 years with a BMI >25 kg/m^2^ were considered eligible. Other than that, exclusion criteria were identical with those described for the 3-week nutrition protocols. Nutritional counseling and calculation of basal metabolic rates were also performed as described above.

Again, blood ketone body concentrations were closely monitored (Glucomen *Aero* 2K; Berlin Chemie AG, Berlin, Germany). During the first week of the diet, daily measurements were performed. A ketogenic state (BHB >0.5 mmol/L) was expected to be achieved latest on the beginning of week 2 (day 8). Thereafter, monitoring was reduced to weekly controls of the blood BHB concentrations. In case of BHB values <0.5 mmol/L, the participant was re-advised based on analysis of food diaries. If BHB levels remained <0.5 mmol/L in the consecutive measurement, the respective participants were excluded from the study. Baseline characteristics of the participants following a three-month KD are shown in Table 4.

### Method details

#### Bioelectrical impedance analysis

A Tanita MC-780 was used for bioimpedance analysis applying a multifrequency approach (5 kHz/50 kHz/250 kHz) according to the manufacturer’s instructions and as guidelined by the European Society of Parenteral and Enteral Nutrition (ESPEN).^39^ Weighing was performed barefoot with only light clothing. During the measurements, study participants remained in an orthostatic position. Conditions prior to bioelectrical impedance analysis were 1) fasting state, 2) no intense physical activity during the last 24 h, 3) no alcohol during the last 24 h, 4) passing urine immediately prior to test. Analyses were performed between 8 a.m. and 10 a.m. After determination of body weight, participants grasped the handles to enable segmental impedance analysis. Thereby, phase angle is being measured and various parameters of body composition are being determined, comprising fat fraction, fat mass, fat free mass, body water, muscle mass, bone mass, visceral fat level, extra- and intracellular water. To calculate BMI, height was determined using a Soehnle Professional 5003 according to the manufacturer’s instructions.

#### Analysis of serum metabolic parameters

Glucose and urea concentrations were quantified by kinetic colorimetric assays using the UREAL (Roche Diagnostics, Cat.# 05171873 190) and GLUC3 kit (Roche Diagnostics, Cat.# 05168791 190). Measurement of cholesterol, triglycerides and lipoproteins was performed via enzymatic color assays using the CHOL2 (Roche Diagnostics, Cat.# 05168538 190), TRIGL (Roche Diagnostics, Cat.# 05171407 190), HDLC4 kit (Roche Diagnostics, Cat.# 07528582 190) and LDLC3 kit (Roche Diagnostics, Cat.# 07005768 190). C-reactive protein concentration was measured by a particle enhanced turbidimetric immunoassay using the CRP4 kit (Roche Diagnostics, Cat.# 07876424 190). A Cobas 8000/c702 system (Roche Diagnostics) was used for these analyses according to the instructions of the manufacturer.

For measurements of apolipoproteins a/b, turbidimetric immunoassays were used (APOAT/APOBT kit, Roche Diagnostics, Cat# 03032566 122/03032574 122) on a Cobas 8000/c502 system (Roche Diagnostics) as to the manufacturer’s instructions.

HbA1c was quantified by high-performance liquid chromatography (HPLC) using the Variant II Turbo HvA1c kit-2.0 (Bio Rad, Cat.# 270-2455EX) on a Variant II Turbo hemoglobin test system (Bio Rad) according to the instructions of the manufacturer.

#### World health Organization’s quality of life assessment (WHOQOL-BREF)

The WHO defines quality of life as the subjective perception of a person’s life situation in the context of culture and value system in relation to objectives, expectations, norms and concerns.^40^ The WHOQOL-BREF is the 26-item abbreviated version of the WHOQOL-100 for assessing quality of life. The questionnaire is completed by the participant and consists of four domains: "Physical health", "Mental health", "Social relationships" and "Environmental quality". For each subscale, a final score is calculated, ranging from 0 to 100, where 0 represents the worst possible condition, and 100 the best.

#### Short form health survey (SF-36)

The SF-36/RAND-36 is a measurement instrument for assessing the health-related quality of life of patients.^41^ Consisting of eight domains, it assesses vitality, physical functioning, bodily pain, general health perceptions, physical role functioning, emotional role functioning, social role functioning and mental health. For evaluation, all answers are first converted into predefined point scales using a scoring key, and the average value is calculated for each domain The total score for each SF-36 subscale ranges from 0 to 100, with a lower score indicating greater functional impairment.^42^

#### Fatigue Assessment Scale (FAS)

Fatigue severity was assessed using the Fatigue Assessment Scale (FAS), an unidimensional fatigue questionnaire, validated in representative population samples,^43^^,^^44^ with a 10-item self-report scale assessing both physical and mental symptoms of fatigue, that enables a short and simple assessment of fatigue and is strongly supported in medical practice.^44^^,^^45^ The FAS total score ranges from 10 to 50, with a higher score indicating more severe fatigue. Scores below 22 represent a healthy state, while scores between 22 and 34 indicate mild to moderate fatigue. Individuals with a score above 35 display severe fatigue.^45^

### Quantification and statistical analysis

The data presented here is part of a prospective nutritional intervention study for which changes in mRNA expression of IFNγ in T cells was set as the primary endpoint (German Clinical Trials Register; DRKS-ID: DRKS0002799). We expected an effect size (cohen’s d) of 1.0, based on BHB blood concentrations of previous results.^9^ Using the paired t-test to compare BHB levels prior to the diet and at the end of the nutritional intervention, with a significance level of α = 0.05 and assuming a dropout rate of 30% as a safety margin, a sample size of 30 subjects following a KD for inclusion was considered appropriate to achieve a power of 95%. Since no dropouts were assumed for the Western diet, this control group comprised a total of 20 volunteers.

If not stated otherwise, statistical analysis was performed using GraphPad Prism 10 (GraphPad Software, Inc., United States). All datasets were analyzed for normal distribution using the Kolmogorov-Smirnov and D'Agostino & Pearson tests. Paired t-test or Wilcoxon matched-pairs signed rank test, as appropriate, served for comparisons. To evaluate whether the level of blood ketone bodies correlate to potential metabolic changes, we calculated Pearson correlation coefficients. To account for multiple testing, the Benjamini Hochberg correction was applied. Statistical significance was assumed at a *p*-value of *p* < 0.05 and an adjusted *p*-value (FDR) < 10%, with ∗*p* < 0.05, ∗∗*p* < 0.01, ∗∗∗*p* < 0.001 and ∗∗∗∗*p* < 0.0001. Biological replicates and data presentations are being specified in the figure legends.

### Additional resources

German Clinical Trials Register; DRKS-ID: DRKS00027992, accessible via https://drks.de/search/de/trial/DRKS00027992.
