# Supplementary material for: The impact of a ketogenic diet on weight loss, metabolism, body composition and quality of life
Source: iScience. 2024 Oct 30;27(12):111291. doi: 10.1016/j.isci.2024.111291 (PMC11612819; doi:10.1016/j.isci.2024.111291)
Supplement: Document S1. Figure S1, Tables S1–S6 and Data S1 and S2 [file mmc1.pdf]

iScience, Volume *27*

Supplemental information

The impact of a ketogenic diet on weight loss, metabolism, body composition and quality of life

Simon Hirschberger, David Effinger, Polina Yoncheva, Annika Schmid, Mara-Noel Weis, Lesca-Miriam Holdt, Daniel Teupser, and Simone Kreth

Supplemental Information

Supplemental Figures

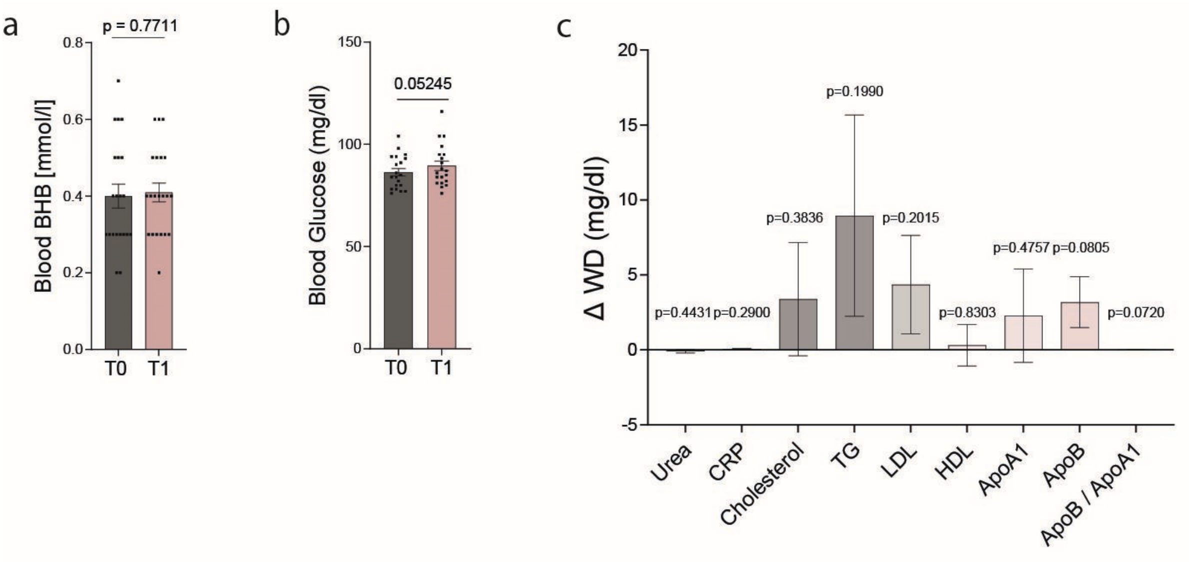

Supplemental Figure 1: Metabolic analysis of three weeks WD control diet. a Blood ketone body concentration quantified via point-of-care blood ketone strips and **b** fasting blood glucose prior to the start of the diet (T0) and at the end of the diet (T1). **c** Changes to metabolic parameters (T1-T0) as indicated. Data depicted as mean ± SEM (a/b) and change (mg/dl) T1-T0 (c), with dots indicating individual values. n = 20. P-values as depicted.

Supplemental Tables

Supplemental Table S1: Physical activity level

Category	PAL value
Sedentary or light activity lifestyle	1.40-1.69
Active or moderately active lifestyle	1.70-1.99
Vigorous or vigorously active lifestyle	2.00-2.40*
Regular competitive sports	> 2.40

Based on the comprehensive anamnesis, the average activity levels of the participants were assessed using the Physical Activity (PAL) according to https://www.fao.org/3/y5686e/y5686e07.htm

Supplemental Table S2: Pearson correlation analysis of 3wKD

	3wKD	
	**Pearsons r**	**p-Value**
BHB T1 to ΔHDL	-0.3333	0.1765
BHB T1 to ΔUrea	0.4019	0.0880
BHB T1 to ΔBMI	-0.1418	0.5510
ΔBMI to ΔHDL	0.05162	0.8388
ΔBMI to ΔCRP	-0.2601	0.2680
ΔBMI to ΔTriglycerides	-0.2297	0.3299
ΔBMI to ΔUrea	-0.3547	0.1249

Delta BMI = T0 BMI - T1 BMI; Delta HDL = T0 HDL - T1 HDL; Delta CRP = T0 CRP - T1 CRP; Delta Urea = T0 Urea - T1 Urea; Delta Triglycerides = T0 Triglycerides - T1 Triglycerides.

Supplemental Table S3: Baseline characteristics of participants on three-weeks WD

	**WD control**
**n**	20
**Age [years],** ***mean ± SD***	41.00 ± 21.34
**Sex [%], male/female/diverse**	45/55/0
**BMI, [kg/m2], mean ± SD**	24.21 ± 4.95
**PAL,** ***mean ± SD***	1.71 ± 0.16
**Calculated caloric intake [kcal/day],** ***mean ± SD***	2564.52 ± 748.49
**Calculated carbohydrate intake [g/day],** ***mean ± SD***	386.89 ± 109.90

Supplemental Table S4: Body composition analysis of participants on a three-week WD

	WD control		
	**T0**	**T1**	**p-value**
**n**	20	20	
**BMI [kg/m^2]**	22.71 ± 0.55	22.67 ± 0.53	0.7051
**Fat fraction [%]**	21.11 ± 1.37	21.09 ± 1.42	0.9486
**Fat mass [kg]**	15.22 ± 1.22	15.22 ± 1.24	0.9768
**Fat free mass [kg]**	56.68 ± 2.25	56.92 ± 2.38	0.4232
**Phase angle [°]**	5.985 ± 0.16	6.026 ± 0.16	0.3427
**Total body water [kg]**	38.89 ± 1.65	39.04 ± 1.68	0.2060
**Predicted muscle mass [kg]**	53.90 ± 2.28	54.10 ± 2.40	0.4928
**Bone mass [kg]**	2.85 ± 0.11	2.86 ± 0.11	0.7492
**Visceral fat mass [kg]**	8.25 ± 0.69	8.26 ± 0.71	0.9686
**Visceral fat level**	3.82 ± 0.60	3.77 ± 0.59	>0.9999
**Extracellular water [kg]**	16.56 ± 0.62	16.57 ± 0.65	0.4303
**Intracellular water [kg]**	22.53 ± 1.08	22.69 ± 1.07	0.1258

Supplemental Table S5: Assessment of health-related quality of life via WHOQOL-BREF and SF-36 questionnaire in participants on a three-week WD

	WD control	p-value
**FAS (mean ± SEM); n=20**		
- Fatigue Assessment Scale	+3.52% % ± 1.90 %	0.0697
**SF-36 (mean ± SEM); n=20**		
- Physical functioning	+0.25 % ± 0.78 %	>0.9999
- Physical role functioning	+3.95 % ± 4.39 %	0.7500
- Emotional role functioning	+1.89 % ± 4.31 %	>0.9999
- Bodily pain	+4.01 % ± 2.58 %	0.1953
- Mental health	-0.65 % ± 2.88 %	>0.9999
- Social role functioning	+1.30 % ± 2.09 %	0.7500
- General health perception	+5.00 % ± 3.84 %	0.3047
- Vitality	+1.48 % ± 4.55 %	0.8791
**WHOQOL-BREF (mean ± SEM); n=20**		
- Physical health	+2.96 % ± 1.82 %	0.1206
- Mental health	-1.51 % ± 1.72 %	0.3881
- Social relationships	+0.37 % ± 2.42 %	0.7148
- Environmental quality	+3.39 % ± 1.65 %	0.0555

Supplemental Table S6: Pearson correlation analysis of 3mKD

3mKD		
BHB T1 to ΔHDL	0.1840	0.4123
BHB T1 to ΔUrea	0.1640	0.4439
BHB T1 to ΔBMI	0.03599	0.8705
ΔBMI to ΔHDL	0.1819	0.4301
ΔBMI to ΔCRP	-0.1753	0.5009
ΔBMI to ΔTriglycerides	0.2948	0.1619
ΔBMI to ΔUrea	-0.2871	0.1737

Delta BMI = T0 BMI - T1 BMI; Delta HDL = T0 HDL - T1 HDL; Delta CRP = T0 CRP - T1 CRP; Delta Urea = T0 Urea - T1 Urea; Delta Triglycerides = T0 Triglycerides - T1 Triglycerides.

Supplemental Methods

Data S1: Nutritional counseling of WD control group, related to STAR methods

The control group was guided to adhere to the ten dietary guidelines for a wholesome diet according to the German Council for Nutritional Medicine (DGE), accessible via https://www.dge.de/gesunde-ernaehrung/dge-ernaehrungsempfehlungen/10-regeln/en/.

1. Enjoy food diversity: Make use of the diversity of foods and eat versatile. Choose mainly plant-based foods.
2. Vegetables and fruit – take five a day: Enjoy at least 3 portions of vegetables and 2 portions of fruit each day. This could also include legumes such as lentils, chickpeas and beans as well as (unsalted) nuts.
3. Favour whole-grain foods: The whole-grain varieties of cereal products like bread, pasta, rice and flour are the best choice for your health.
4. Complete the choice with animal-based foods: Consume milk and dairy products such as yogurt and cheese daily, fish once to twice a week. If you eat meat, you should not consume more than 300 to 600 g per week.
5. Choose health-promoting fats: Prefer vegetable oils like rapeseed oil and margarines produced therefrom. Avoid hidden fats.
6. Reduce sugar and salt intake: Sugar-sweetened foods and beverages are not recommendable and should be avoided whenever possible. Sugar should only be consumed in small amounts.
7. Water is the best choice: Drink about 1.5 liters per day. Water or other calorie-free beverages, such as unsweetened tea, are the best choice. Sugar-sweetened and alcoholic beverages are not recommended.
8. Prepare carefully cooked dishes: Cook food as long as necessary but as short as possible, using little amount of water and fat. Avoid burning the food during roasting, grilling, baking and frying.
9. Mindful eating and enjoying: Take a break while you eat and allow plenty of time for eating.
10. Watch your weight and stay active: Combine a wholesome diet with plenty of physical activity. It is not only regular exercise which is helpful, but also an active daily life which includes frequent walking and cycling.

Data S2: CONSORT Harms 2022 integrated into CONSORT 2010 items checklist of information to include when reporting a randomised trial

Section/Topic	Item No	Checklist item	Reported on page No
**Title and abstract**			
	1a	Identification as a randomised trial in the title	not applicable
	1b	Structured summary of trial design, methods, results of outcomes of benefits and harms, and conclusions (for specific guidance see CONSORT for abstracts)	2
**Introduction**			
Background and objectives	2a	Scientific background and explanation of rationale	3-4
	2b	Specific objectives or hypotheses for outcomes benefits and harms	3-4
**Methods**			
Trial design	3a	Description of trial design (such as parallel, factorial) including allocation ratio	22-25
	3b	Important changes to methods after trial commencement (such as eligibility criteria), with reasons	not applicable
Participants	4a	Eligibility criteria for participants	23-24
	4b	Settings and locations where the data were collected	22
Interventions	5	The interventions for each group with sufficient details to allow replication, including how and when they were actually administered	22-25 + Supplement
Outcomes	6a	Completely defined pre-specified primary and secondary outcome measures for both benefits and harms, including how and when they were assessed	22
	6b	Any changes to trial outcomes after the trial commenced, with reasons	not applicable
	6c	Describe if and how non-prespecified outcomes of benefits and harms were identified, including any selection criteria, if applicable	not applicable
Sample size	7a	How sample size was determined	27
	7b	When applicable, explanation of any interim analyses and stopping guidelines	not applicable

Section/Topic	Item No	Checklist item	Reported on page No
Randomisation:			
Sequence generation	8a	Method used to generate the random allocation sequence	not applicable
	8b	Type of randomisation; details of any restriction (such as blocking and block size)	not applicable
Allocation concealment mechanism	9	Mechanism used to implement the random allocation sequence (such as sequentially numbered containers), describing any steps taken to conceal the sequence until interventions were assigned	not applicable
Implementation	10	Who generated the random allocation sequence, who enrolled participants, and who assigned participants to interventions	not applicable
Blinding	11a	If done, who was blinded after assignment to interventions (e.g., participants, care providers, those assessing outcomes of benefits and harms) and how	not applicable
	11b	If relevant, description of the similarity of interventions	not applicable
Statistical methods	12a	Statistical methods used to compare groups for primary and secondary outcomes of both benefits and harms	27
	12b	Methods for additional analyses, such as subgroup analyses and adjusted analyses	not applicable
**Results**			
Participant flow (a diagram is strongly recommended)	13a	For each group, the numbers of participants who were randomly assigned, received intended treatment, and were analysed for outcomes of benefits and harms	5-7
	13b	For each group, losses and exclusions after randomisation, together with reasons	5-7
Recruitment	14a	Dates defining the periods of recruitment and follow-up for outcomes of benefits and harms	22
	14b	Why the trial ended or was stopped	not applicable
Baseline data	15	A table showing baseline demographic and clinical characteristics for each group	18,20, Supplement
Numbers analysed	16	For each group, number of participants (denominator) included in each analysis and whether the analysis was by original assigned groups and if any exclusions were made	5-8, 18-21
Outcomes and estimation	17a	For each primary and secondary outcome of benefits and harms, results for each group, and the estimated effect size and its precision (such as 95% confidence interval)	5-8, 18-21, 27
	17a2	For outcomes omitted from the trial report (benefits and harms), provide rationale for not reporting and indicate where the data on omitted outcomes can be accessed	not applicable
	17b	Presentation of both absolute and relative effect sizes is recommended, for outcomes of benefits and harms	not applicable
	17c	Report zero events if no harms were observed	5-7
Ancillary analyses	18	Results of any other analyses performed, including subgroup analyses and adjusted analyses, distinguishing pre-specified from exploratory	not applicable
Harms	19	All important harms or unintended effects in each group (for specific guidance see CONSORT for harms)	not applicable

Section/Topic	Item No	Checklist item	Reported on page No
**Discussion**			
Limitations	20	Trial limitations, addressing sources of potential bias related to the approach to collecting or reporting data on harms, imprecision, and, if relevant, multiplicity or selection of analyses	12
Generalisability	21	Generalisability (external validity, applicability) of the trial findings	9-12
Interpretation	22	Interpretation consistent with results, balancing benefits and harms, and considering other relevant evidence	9-12
**Other information**			
Registration	23	Registration number and name of trial registry	2, 13
Protocol	24	Where the full trial protocol and other relevant documents can be accessed, including additional data on harms	2, 13
Funding	25	Sources of funding and other support (such as supply of drugs), role of funders	13

Note: Adapted from Schulz (2010) to integrate items of CONSORT Harms 2022 (Junqueira 2022) [https://creativecommons.org/licenses/by/2.0/]. CONSORT items 1b, 2b, 6a, 11a, 12a, 13a, 14a, 16a, 17a, 17b, 18, 20 and 24 of were modified to incorporate elements relevant to the reporting of harms. Two new items were added (item 6c and 17a2). Please see the CONSORT Harms 2022 statement for additional details (Junqueira 2022).

We strongly recommend reading the CONSORT 2010 statement (Schulz 2010) in conjunction with the CONSORT Harms 2022 statement (Junqueira 2022) for important clarifications on all the items. If relevant, we also recommend reading CONSORT extensions for cluster randomised trials, non-inferiority and equivalence trials, non-pharmacological treatments, adaptive designs, pilot and feasibility studies, multi arm trials, cross-over and pragmatic trials. Additional extensions are forthcoming: for those and for up-to-date references relevant to this checklist, see EQUATOR Network.

References

Junqueira DR, Zorzela L, Golder S, Loke Y, Gagnier JJ, Julious SA, Li T, Mayo-Wilson E, Pham B, Phillips R, Santaguida P, Scherer RW, Gøtzsche PC, Moher D, Ioannidis JPA and Vohra S on behalf of the CONSORT Harms Group. CONSORT Harms 2022 statement, explanation, and elaboration: updated guideline for the reporting of harms in randomised trials. *BMJ* 2023 **381:** e073725 DOI 10.1136/bmj-2022-073725

Schulz KF, Altman DG, Moher D, for the CONSORT Group. CONSORT 2010 Statement: updated guidelines for reporting parallel group randomised trials. *BMJ* 2010 **340:**c332 doi: 10.1136/bmj.c332.
